# Testing a Cognitive Control Model of Human Intelligence

**DOI:** 10.1038/s41598-019-39685-2

**Published:** 2019-02-27

**Authors:** Yu Chen, Alfredo Spagna, Tingting Wu, Tae Hyeong Kim, Qiong Wu, Caiqi Chen, Yanhong Wu, Jin Fan

**Affiliations:** 1 Department of Psychology, Queens College, The City University of New York, Queens, NY USA; 2 Department of Psychology, The Graduate Center, The City University of New York, New York, NY USA; 3 Department of Psychology, Columbia University in the City of New York, New York, NY USA; 4 Physiological Investigations of Clinically Normal and Impaired Cognition Laboratory, Institute du Cerveau et de la Moelle Epiniere, Paris, France; 5 School of Psychology, Capital Normal University, Beijing, China; 6 School of Psychology, South China Normal University, Guangzhou, Guangdong, China; 7 School of Psychological and Cognitive Sciences, Peking University, Beijing, China; 8 Beijing Key Laboratory of Behavior and Mental Health, Peking University, Beijing, China; 9 Key Laboratory of Machine Perception (Ministry of Education), Peking University, Beijing, China

## Abstract

The definition of human intelligence and its underlying psychological constructs have long been debated. Although previous studies have investigated the fundamental cognitive functions determining intellectual abilities, such as the broadly defined executive functions including working memory, the core process has yet to be identified. A potential candidate for such a role might be cognitive control, a psychological construct for the coordination of thoughts and actions under conditions of uncertainty. In this study, we tested a cognitive control model of intellectual ability by examining the association between cognitive control, measured by a perceptual decision-making task and by the attention network test, and general intelligence including components of fluid intelligence (Gf, concerning the ability to solve problems by abstraction) and crystalized intelligence (Gc, related to learning from prior knowledge and experience) measured by the Wechsler Adult Intelligence Scale. We also examined the potential role of cognitive control as a core process involved in another determinant of intellectual abilities, the working memory, measured by the N-back tasks and the working memory complex span tasks. The relationship among intelligence, cognitive control, and working memory was examined using structural equation modeling. Results showed that cognitive control shared a large amount of variance with working memory and both measures were strongly associated with Gf and Gc, with a stronger association with Gf than Gc. These findings suggest that cognitive control, serving as a core construct of executive functions, contributes substantially to general intellectual ability, especially fluid intelligence.

## Introduction

Although intelligence has been thought of as the most prominent property that makes humans unique in the history of biological evolution^1,2^, the challenges associated with capturing its ultimate nature^3^ have had a significant impact on the consensus regarding its definition. The early attempt to define intelligence was conducted by Charles Spearman^4^, who hypothesized the existence of a general factor, *g*, as the core of all cognitive abilities. This unitary conception of intelligence has, however, been challenged by a variety of models of intelligence, including the Primary Mental Abilities^5^, the Structure of Intellect^6^, and the Theory of Multiple Intelligences^7^, with all of them proposing that intelligent behavior arises from a collection of factors, e.g., verbal comprehension, spatial visualization, reasoning, and processing speed. These intellectual abilities have been further synthetized as two components, the fluid intelligence (Gf), reflecting the ability to solve problems by abstraction and supported by the multiple-demand system in the brain^8,9^, and the crystallized intelligence (Gc), concerning the ability to learn from previous knowledge, with *g* located at the apex of this hierarchical model^10–12^. Most of these theories were derived from a psychometric approach and aimed at quantifying this psychological phenomenon, but this approach has been extensively challenged^13–16^ and the process(es) underlying the *g* factor remains unclear.

In contrast to looking for a unique component of intelligence, the triarchic theory of intelligence^16^ defines it as comprising three components: the metacomponents, the performance components, and the knowledge-acquisition components. The metacomponents refer to the executive processes involved in problem solving, including mental manipulation and management. The performance components work as the carrier to implement the outcome of metacomponents, i.e., carrying out the actions. The knowledge-acquisition components are associated with the mental processes to obtain new information involving selectively dealing with relevant information and combining various pieces of information^16^. To some extent, the existence of a common element of information processing among these three components is indicated^17^, but the nature of this process remains unclear. More recently, the Planning, Attention-Arousal, Simultaneous and Successive (PASS) theory of intelligence^13–15^ suggested that intelligence is implemented across a variety of domains and consists of interdependent, but separate, functions supported by different brain areas. Specifically, the process of planning involves executive functions to control and organize behaviors by selecting and constructing strategies, and monitoring performance; the attention-arousal process requires maintaining arousal levels and alertness, and selectively focusing on relevant information; the simultaneous processing and successive processing are responsible for encoding, transforming, and recollecting information. Both the triarchic theory of intelligence and the PASS theory constitute the attempts to embrace both qualitative and quantitative perspectives, and to emphasize the mental processes and operations involved in the intellectual behaviors. Although contemporary theories, whether psychometric or cognitive, have attempted to define intelligence in terms of different components, it remains unclear whether a unique component is at the basis of these functions, leaving the question about the core process of intelligence open.

In an effort to solve this puzzled picture, prior work has proposed that working memory, a cognitive function comprising temporary storage spaces entangled with a central executive component in charge of the manipulation of stored information^18–20^, might be the psychological core of the Gf ^21–23^. Although evidence of a strong relationship between measures of working memory capacity and of the Gf ^23–27^ exists, this result has been shown to reside on the shoulder of the central executive component only, while the storage component does not seem to correlate with the Gf ^28^. This evidence suggests that it is the central executive component^20,29^ that plays a key role in the relationship between working memory and the Gf. Another psychological construct that has frequently been related to the Gf is the broad “executive functions”, which can be divided into three sub-functions of updating, inhibiting, and shifting^30,31^. Updating refers to the ability to dynamically manipulate the contents of working memory, and it can be measured by the N-back task^32^, inhibiting is for the suppression of inappropriate responses, and it can be measured by the Stroop task^33^, and shifting is the switch between multiple tasks, operations, or mental sets, and it can be measured by the category switch task^34^. While the updating component has been shown to be strongly related to the Gf ^30,35^, the weaker to non-significant association between inhibiting/shifting and the Gf ^30,35–37^ may result from the fact that the tasks (e.g., the Stroop task and the category switch task) used in these studies may have been tapping less on the core of the executive functions^34,38^. We speculate that a stronger relationship between the executive functions and the Gf would be identified if tasks that require a greater extent of mental coordination, which is the function of cognitive control, were used to examine executive functions. In the past hundred years, a variety of tests were developed to capture the essence of intelligence, with more recent efforts devoted to understanding the role of abstract reasoning in the Gf ^16,39,40^. Abstraction has been defined as a solution for the new knowledge paradox^41,42^ emerging gradually from the coordination of the thoughts^43^, a definition that closely resembles that of cognitive control, a high-level mental operation required to coordinate thoughts and actions under conditions of uncertainty^44–47^. Cognitive control may serve as the core component of both working memory and executive functions and the significant relationship between working memory/updating function and the Gf should be accounted by the common involvement of cognitive control.

The present study aimed to advance the current understanding of the role of cognitive control in human intelligence. We examined the relationship between cognitive control and intellectual ability, as well as the relationship between cognitive control and working memory in light of their relationship with intellectual abilities. If working memory and cognitive control are two independent cores of intelligence, their measurements should be highly correlated to intellectual abilities, but not with each other. Alternatively, if cognitive control and working memory share a common process, as the core of intelligence, their estimates should share a large amount of variance and therefore should be significantly correlated, as well as correlated to the measures of intellectual abilities. We proposed that the latter case would be true, and predicted that the shared component between these constructs would be related to the coordination of thoughts and actions, rather than memory. Further, cognitive control should be more related to the construct of the Gf, rather than the Gc because the function of mental coordination is at the core of the Gf.

## Material and Methods

### Participants

Students who were taking the General Psychology (Psych101) course at Queens College, the City University of New York, participated in this study (n = 151) and received academic course credits for their participation. The study was approved by the Institutional Review Board of Queens College of the City University of New York, all research was performed in accordance with relevant guidelines/regulations, and written informed consent was obtained from each individual prior to participation. In order to rule out the influence of different languages on the estimation of intellectual abilities^48^, participants with less than 15 years of education in the United States (n = 41) were considered non-native speakers and excluded from further data analysis. An additional 22 participants were also excluded from further analysis due to the following performance-related reasons: (1) the proportion of trials with valid responses across all conditions was less than 95% in the Majority Function Task - Masked (n = 8); (2) the overall response accuracy in the Attention Network Test – Revised (n = 4), or in the 0-back condition in any of the N-back tasks was lower than 90% (n = 5); (3) the response accuracy in the distracting task in any of the working memory complex span tasks was lower than 85% (n = 5). The final sample size consisted of 88 participants (53 females; age: 18–31 years; mean age: 20.42 years, SD = 2.88).

### Measurement of intellectual ability

#### Wechsler adult intelligence scale - fourth edition (WAIS-IV)

The WAIS-IV^49^ was used to obtain a comprehensive assessment of participants’ general intellectual abilities through the administration of the following ten core subtests: Block Design (assemble red-and-white blocks based on a given model-picture within time constraint), Similarities (summarize how two objects or concepts are similar), Digit Span (recall numbers in a forward order, a backward order, and in an ascending order), Matrix Reasoning (select a comparable picture for the incomplete matrix or pattern), Vocabulary (define words), Arithmetic (mentally solve arithmetical problems within time constraint), Symbol Search (make decision on whether a set of items matches with sample items within time constraint), Visual Puzzle (select components to reconstruct a given puzzle within time constraint), Information (answer questions regarding general knowledge), and Coding (copy symbols based on a key template within time constraint).

Four composite scores representing major components of intellectual abilities were derived from the following combinations of subtests^50,51^: (1) Verbal Comprehension Index (VCI), derived from the subtests of Similarities, Vocabulary, and Information, to represent the ability of verbal reasoning and accumulated knowledge from learning and education; (2) Perceptual Reasoning Index (PRI), derived from the subtests of Block Design, Matrix Reasoning, and Visual Puzzles, to represent the ability of abstract perceptual reasoning and spatial processing; (3) Working Memory Index (WMI), derived from the subtests of Digit Span and Arithmetic, to represent the ability of mentally manipulation on information stored temporarily in memory; and (4) Processing Speed Index (PSI), derived from the subtests of Symbol Search and Coding, to represent the ability of fluently process information, including visual-motor coordination and cognitive decision-making. The Full Scale Intelligence Quotient (FSIQ) was based on the total combined performance of VCI, PRI, WMI, and PSI, which estimates the general intellectual ability.

Consistent with prior work that interpreted VCI as a measurement of verbal ability, comprehension, knowledge, and crystalized intelligence^52^, we categorized VCI as the manifest variable of the Gc construct in the structural equation modeling (SEM). According to the description of composite scores in the technical and interpretive manual of WAIS-IV^53^, PRI represents the ability of perceptual and fluid reasoning, spatial processing, and visual-motor integration, WMI characterizes the ability of temporary maintenance of information in memory while performing mental operation on it, and PSI indicates the ability of simple visual information processing, short-term visual memory, attention, and visual-motor coordination. These three composite scores (PRI, WMI, PSI) are related to the nature of the Gf, i.e., coordination of mental resources to solve abstract, novel problems^54^, and were categorized as the manifest variables of the Gf in the SEM.

### Measurements of cognitive control

To measure cognitive control, we used two tasks that are theoretically independent of short- and long-term memory requirements: the backward Majority Function Task-Masked^46^ (MFT-M) and the Attention Network Test-Revised (ANT-R)^55,56^. The MFT-M estimates the capacity of cognitive control (CCC) by purportedly challenging an individual’s upper limit of information processing. The ANT-R^55,56^ measures the processing efficiency of cognitive control by the flanker conflict effect^57^.

#### The backward majority function task-masked (MFT-M)

**Figure 1:**
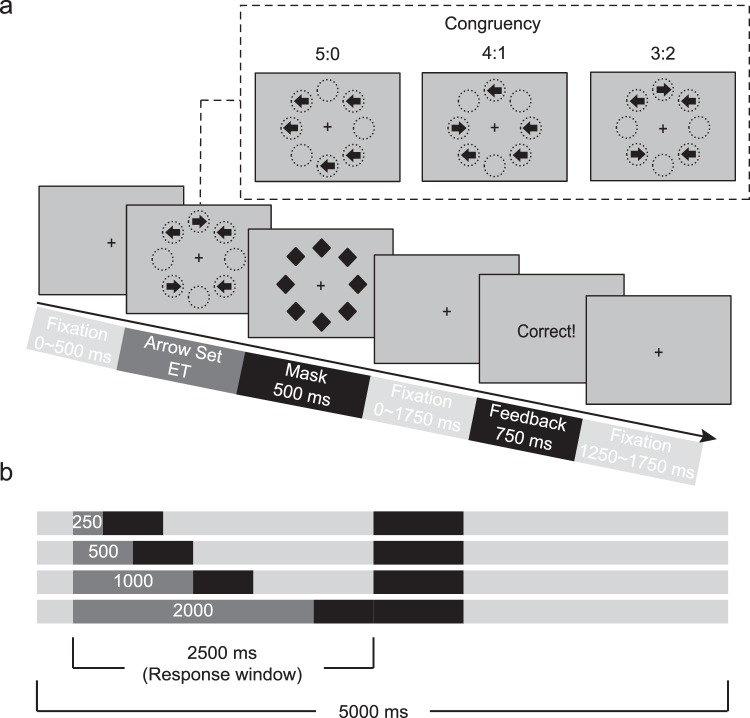
Schematic of the backward majority function task-masked (MFT-M). (**a**) An illustration of a 3:2 congruency condition in the MFT-M and the event sequence of a trial. Participants are requested to report the majority of arrow directions (left or right) among a set of imperative stimuli. The upper-right panel shows the three possible arrow ratios. (**b**) Timeline of the stimuli presentation under different exposure time (ET, in ms). Duration of each event is indicated by the length of each texture. Participants are required to make a response within a 2500 ms response window starting from the onset of the arrow set and the entire trial lasts 5000 ms.

We used a modified version of the MFT-M that uses only one condition of arrow set size, i.e., five arrows as the stimuli, in order to maintain the total task duration to less than 1 hour. Other parameters were kept identical to the original version. In each trial (Fig. 1a), after a variable fixation period ranging between 0 and 500 ms, a set of five arrows was displayed simultaneously and randomly distributed at eight possible locations arranged as an octagon centered on a fixation cross, with each arrow pointing either left or right. The arrow congruency (the number of arrows pointing to the majority direction vs. the number of arrows pointing to the minority direction) could be 5:0, 4:1, or 3:2. After a varied exposure time (ET) of 250, 500, 1000, or 2000 ms **(**Fig. 1b**)**, the arrow set was replaced by a mask of eight diamonds at the same eight possible locations, and the mask set was presented for 500 ms. Subsequently, an after-mask fixation interval ranging between 0 and 1750 ms was presented, making the total duration of the response window 2500 ms. Participants had to make a response by clicking either the left or right mouse button to indicate the majority direction of the arrows presented in the set of imperative stimuli as accurately and quickly as possible. Responses were required to be made in all trials, with participants being instructed to guess if they were unsure about the correct answer, within a fixed response window of 2500 ms starting from the onset of the arrow set. After the response window, a feedback indicating whether the response was correct or not was shown for 750 ms, followed by a fixation period of 1250–1750 ms. Each trial lasted 5000 ms in total. The experimental design was, therefore, a 3 (Congruency) × 4 (ET) factorial design. For each block, there was only one ET condition presented in combination with three conditions of Congruency. The task comprised of 12 blocks (3 blocks for each ET) presented in a random order, and each block consisted 36 trials, for a total of 432 trials for the task. The total duration of the task was approximately 40 minutes.

Response accuracy for each condition, calculated as the percentage of correct trials, was used to estimate the CCC. In our previous studies^46,58,59^, we demonstrated that the grouping search algorithm is the most plausible strategy used in the Majority Function Task. With this strategy, participants keep randomly sampling 3 arrows as a group (majority size, *N*_*maj*_) out of 5 (total set size) until acquiring a congruent sample, i.e., all three arrows in the sampled group are pointing to the same direction. The average numbers of to-be-scanned arrows (*N*) is computed as *N*_*maj*_ divided by the probability of obtaining a congruent group from sampling attempts (*P*_*group*_). Corresponding to this strategy, the information entropy of each congruency condition is calculated as the *log*_2_ transformation of *N*, resulting in 1.58 bits for the 5:0 condition, 2.91 bits for the 4:1 condition, and 4.91 bits for the 3:2 condition, respectively. The information rate of each *ET* condition (in the unit of bits per second, *bps*) is calculated as the *log*_2_ transformation of *N* per second, i.e., *log*_2_ (*N*/*ET*), resulting in a range of information rate between 0.59 bps and 6.91 bps. The CCC of each participant refers to the maximum information rate that can be reliably processed. An individual with a high CCC can reliably handle high-level mental operations under time constraints, and therefore should perform better in tasks requiring cognitive control.

Due to the influence of time constraint, i.e., the *ET*, in responding to the arrow set in each trial, the sampling process can be categorized as either voluntarily terminated (VT) or forcefully terminated (FT). For the VT trials, a response is made when a congruent sample is acquired, which leads to a correct response. Higher CCC, longer ET, and lower information entropy will result in a greater probability of VT trials. The response accuracy on the VT trial depends on the baseline response accuracy (*p*_0_) that can be computed as the average accuracy across all congruent conditions (i.e., the arrow congruency of 5:0). While for the FT trials, response is made by guessing because the arrow set disappears before a congruent sample is acquired, which leads to a random response. The probability of guessing correctly is at chance level (*p*_*guess*_ = 0.5). The expected response accuracy (*E* [accuracy]) is computed as the sum of response accuracy on the VT and FT trials using the equation below in which *C* is a free parameter denoting the CCC. Details and derivations of this equation have been shown in our previous study^46^.

$$\begin{array}{rcl}E\,[accuracy] & = & [1-{(1-{P}_{group})}^{\frac{{2}^{C}\times ET}{{N}_{maj}}}]\times {p}_{0}+{(1-{P}_{group})}^{\frac{{2}^{C}\times ET}{{N}_{maj}}}\times {p}_{guess}\\  & = & {p}_{0}-{(1-{P}_{group})}^{\frac{{2}^{C}\times ET}{{N}_{maj}}}\times ({p}_{0}-{p}_{guess}).\end{array}$$

Response accuracy in each condition can be predicted for a given parameter *C*. The CCC can be estimated as the *C* value that provides optimal likelihood between the predicted and the empirical response accuracy across all conditions.

#### The attention network test-revised (ANT-R)

**Figure 2:**
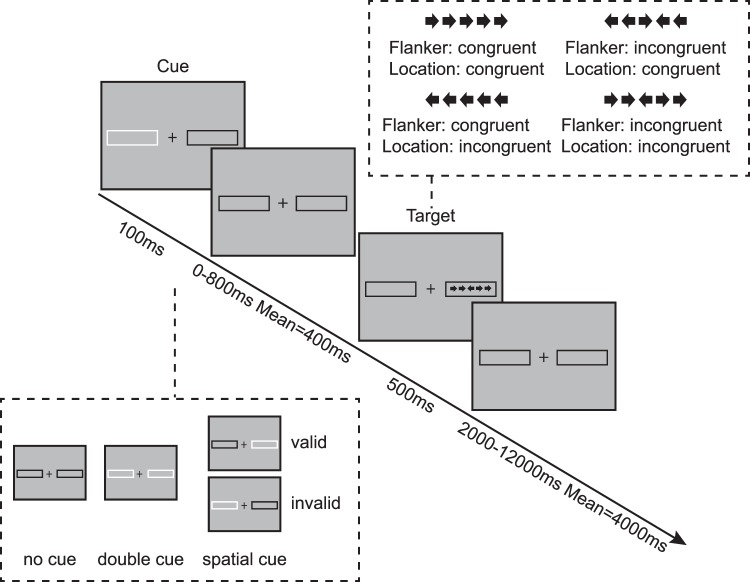
Schematic of the revised attention network test (ANT-R). For each trial, a 100 ms cue (no box flashes under the no cue condition, a single box flashes under the valid and invalid cue conditions, and both boxes flash under the double cue condition) is followed by a variable fixation period (0, 400, 800 ms) and then by the imperative target. The target (the center arrow) is flanked on each side by two other arrows presented for 500 ms, followed by an inter-trial fixation period varying between 2000 and 12000 ms (mean = 4000 ms). Participants are required to report the direction of the target.

In this task, participants were required to identify the direction of an arrow that was flanked by two other arrows on each side. The flankers could point either in the same direction as the target arrow (congruent condition) or in the opposite direction (incongruent condition) of the target arrow. Target and flankers were presented within one of two boxes located either at the right or at the left side of a central fixation cross (Fig. 2). In each trial, a visual cue in the form of a 100 ms flashing of the contours of the boxes, was displayed 0, 400, or 800 ms before the target. There were four cue conditions: double-cue (both boxes flashed, giving temporal but not spatial information about the upcoming target), valid-cue (one of the two boxes flashed, providing temporal and spatial information about the correct location where the target would appear), invalid-cue (one of the two boxes flashed, selecting the alternative location as opposed to the location where the target would be presented), and no-cue (neither of the boxes flashed prior to the target display). Participants were required to respond as quickly and accurately as possible within 1700 ms from the target onset, by clicking either the left or right button on the mouse. The interval between trials varied from 2000 to 12000 ms (mean = 4000 ms). Each trial lasted about 5000 ms on average. There were 4 blocks consisting of 72 trials in each block, for a total of 288 trials and approximately 30-minute task duration.

Trials with error response or with response time (RT) exceeding ± 3 SD of the mean RT in each condition (congruent, incongruent) were removed from further analysis. In total, 1.25% of trials were excluded. Mean RT in each condition was then calculated based on the remaining trials, and was used to estimate the executive control (EC) function^55^. The conflict effect was calculated by subtracting the mean RT of the congruent condition from the mean RT of the incongruent condition. Typically, a more positive conflict effect suggests lower cognitive control ability. Because we hypothesized that general intelligence would be positively correlated to cognitive control ability, and in order to obtain all positive values of estimates to be included in the SEM, we reversely coded this variable by inverting the terms used in formula, therefore subtracting the mean RT of the incongruent trials from the mean RT of the congruent trials (conflict effect = RT_congruent_ − RT_incongruent_). Thus, a less negative conflict effect indicates higher cognitive control ability. Because the executive control is related to the coordination of thought to guide complex behavior via supramodal mechanisms^45,56^, as indexed by the conflict effect, the EC was included as an additional index of cognitive control in this study.

### Measurements of working memory

N-back tasks (spatial and verbal) and working memory complex span tasks were used to measure different aspects of working memory. The N-back tasks assess the ability of challenging control over familiarity-based responding^60^, or recognition-based discrimination processes^61^. The working memory complex span tasks measure the ability of actively recalling and concurrently processing information^61^.

#### Spatial and verbal N-back tasks

**Figure 3:**
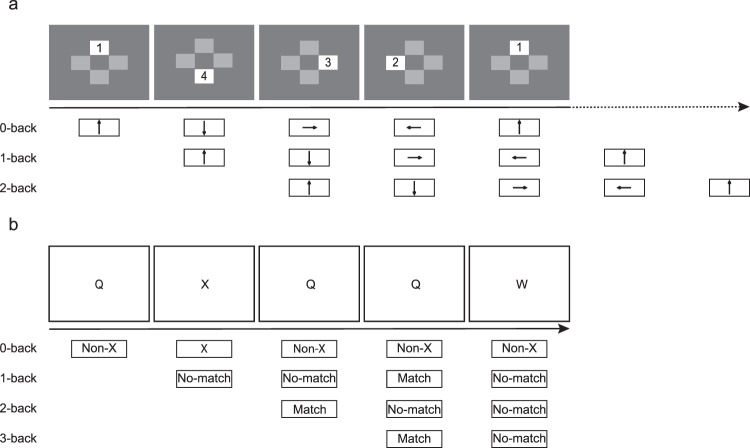
Schematic of the spatial and verbal N-back tasks. (**a**) Illustration of the spatial N-back task. Three conditions are involved in the task: 0-back, 1-back, and 2-back. Participants should make responses to the location of the yellow box each trial (1 represents up, 2 represents left, 3 represents right, and 4 represents down). The arrow under each stimulus indicates the correct response key. (**b**) Illustration of the verbal N-back task. Four conditions are involved in the task: 0-back, 1-back, 2-back, and 3-back. For the 0-back condition, participants should click ‘left’ botton when the letter X is presented, and click ‘right’ botton when other letters are presented. For the other conditions, participants should click ‘left’ button when the current letter matches the letter 1/2/3 times ago, otherwise click ‘right’ button.

Participants completed a spatial and a verbal N-back task sequentially. In the spatial N-back task^62^, four gray boxes were located above, below, to the left, and to the right of a central fixation cross (see Fig. 3a). In each trial, one of the four gray boxes turned yellow for 1538 ms. Participants responded to the location of the yellow box in the 0-back condition, the location of the previous yellow box in the 1-back condition, and the location of the yellow box two trials before in the 2-back condition. Participants responded by pressing the corresponding arrow key on the keyboard. Blocks of the three conditions (0-, 1-, and 2-back) were presented sequentially, and each repeated four times, resulting in a total of twelve blocks. At the beginning of each block, participants were instructed about the upcoming task condition. Each block contained 20 trials and lasted approximately 31 seconds. The total number of trials was 240, and the entire task lasted about 7 minutes.

In the verbal N-back task, a series of letters was presented sequentially for a duration of 1500 ms each, and four conditions (blocks) were presented in a fixed order: 0-back, 1-back, 2-back and 3-back (see Fig. 3b). For the 0-back block, participants were instructed to decide whether the current letter on screen was an ‘X’. For the other blocks, participants were instructed to decide whether the current letter on screen matched the letter presented on one trial before (the 1-back block), on two trials before (the 2-back block), or on three trials before (the 3-back block). Each block consisted of 18 letters. The entire task lasted about 5 minutes.

In both N-back tasks, the response accuracy (ACC) for each condition was calculated. A spatial N-back task index was estimated by subtracting the ACC of the easiest condition (ACC_0-back_) from the ACC of the hardest condition (ACC_2-back_): spatial N-back = ACC_2-back_ − ACC_0-back_. Similarly, the performance index of the verbal N-back task was determined by subtracting the ACC of the easiest condition (ACC_0-back_) from the ACC of the hardest condition (ACC_3-back_) from: verbal N-back = ACC_3-back_ − ACC_0-back_. The ACC of the 0-back condition is expected to be higher than in any other condition, therefore, the indices for both the spatial and verbal N-back performance should be negative. The closer the negative N-back index is to zero, the smaller the difference between the easiest and hardest condition, and the better the performance.

#### Working memory complex span tasks

Participants completed shortened versions of three working memory complex span tasks in sequence: operation span (OSpan), rotation span (RotSpan), and symmetry span (SymSpan)^63^ (http://englelab.gatech.edu/tasks.html). In each task, participants were required to remember a sequence of items (e.g., a sequence of letters in the OSpan task) while completing a distractor task (e.g., solving a math problem) presented between each item in the sequence. Feedbacks including participants’ performance on the current trial (for both the memory task and the distractor task) and the cumulative accuracy were presented on the screen at the end of each trial.

**Figure 4:**
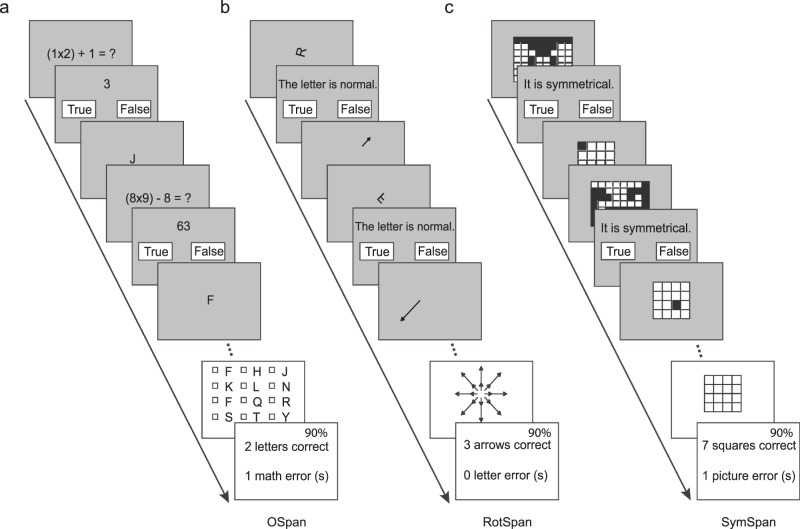
Schematics of the working memory span tasks. (**a**) An illustrative trial of the operational span task (OSpan). A math problem to be solved is followed by a letter. Subsequently, another math problem to be solved is followed by another letter. At the end of the trial, all letters should be recalled by checking corresponding boxes sequentially, followed by a feedback. (**b**) An illustrative trial of the rotation span task (RotSpan). A rotated letter to be judged is followed by an arrow. Subsequently, another rotated letter to be judged is followed by another arrow. At the end of the trial, all arrows should be recalled (both size and direction) by checking corresponding arrowheads sequentially, followed by a feedback. (**c**) An illustrative trial of the symmetrical span task (SymSpan). A picture to be judged as symmetrical or not is followed by a square at one location of the 4 × 4 grid. Subsequently, another picture to be judged is followed by another square at a different location. At the end of the trial, all squares (locations) should be recalled by checking corresponding location boxes sequentially, followed by a feedback.

In each trial of the OSpan task (Fig. 4a), two to seven letters (e.g., “J and F”) appeared sequentially at the center of the screen. A simple math problem (e.g., “(1 × 2) + 1 = ?”) was presented as the distracting task before the presentation of each letter (e.g., J). After all the letters were presented, participants were required to recall all of them in the order presented by sequentially checking the corresponding boxes on the screen. Feedbacks including the number of letters correctly recalled, the number of committed errors in solving the math problems, and the cumulative accuracy of math problems were presented.

In each trial of the RotSpan task (Fig. 4b), three to seven arrows appeared sequentially, being either short or long in length and pointing towards one of eight possible directions. Before the presentation of each arrow, a distracting task was presented, which required participants to judge whether a rotated letter was presented in its normal configuration (e.g., R) or backwards (mirrored). After all of the arrows were presented, participants were asked to recall them with the correct length and direction, and in the order they were presented by sequentially selecting the arrows on the response screen. Feedbacks including the number of arrows with correct length and direction that were successfully recalled, the number of errors regarding letter rotation, and the cumulative accuracy of letter-rotation task were presented.

In each trial of the SymSpan task (Fig. 4c), two to five red squares appeared sequentially and at different locations (one of sixteen possible locations on a 4 × 4 grid). Before the presentation of each square, a distracting task required participants to judge whether a picture was vertically symmetrical or not. At the end of a trial, participants were asked to recall all the locations of red squares in the order presented by sequentially clicking the squares on the response screen. Feedbacks including the number of squares that were correctly recalled, the number of errors regarding symmetrical pictures, and the cumulative accuracy of the picture task were presented.

Participants were required to keep at least 85% accuracy on each distracting task. There were 10 trials in the OSpan, and 8 trials in both the RotSpan and SymSpan. Each task lasted about 10 minutes. The whole section of working memory span tasks lasted about 30 minutes.

The all-or-nothing load (ANL) scoring^64^ was calculated as the ratio between the sum of the correctly recalled elements in correct serial order and the total amount of elements to be recalled in the task, which is ranged between 0 and 1. The ANL is the mostly used method to evaluate the performance in the OSpan, RotSpan, and SymSpan tasks. “All-or-nothing” refers to trials in which all the memory elements were recalled in the correct serial order to be counted as a correct trial, while “load” refers to the response accuracies being weighted by the set size of the memory elements in each trial. Therefore, a higher ANL score indicates a larger working memory span.

### Procedure

All the behavioral tasks were compiled and run on a PC using E-Prime 2.0 software (Psychology Software Tools, Pittsburgh, PA). Participants were required to finish the entire battery of tasks on two separate days within one week. In the first part (day) of the study, each participant first completed three working memory span tasks sequentially: OSpan, RotSpan, and SymSpan. In each span task, they practiced for three trials transitioning from easy to difficult to familiarize with the task, and then continued with the experimental session. After the span tasks, they completed 10 subtests of the WAIS-IV, in a fixed order. Each subtest took approximately 6–8 minutes to complete, for a total duration of approximately 60–80 minutes. In the second part (day) of the study, participants completed the MFT-M, ANT-R, spatial N-back task, and verbal N-back task in a fixed order. For each task, a short practice session was performed before the experimental session. Participants were allowed to rest as long as needed between tasks.

### Data analysis

To examine the relationship among all the measures, one-tailed Pearson’s correlation analyses were conducted. In addition, the Bayes Factor (BF) was calculated for each correlation^65^. A BF greater than 100 indicates decisive evidence for the alternative hypothesis (*H*_1_) that there is a real correlation in the population, a BF greater than 3 suggests substantial evidence for the correlation, while a BF less than 1/3 indicates substantial evidence for the null hypothesis *H*_0_ that there is no correlation in the population, and any BF value ranging from 1/3 and 3 suggests insensitivity of the data to distinguish between the *H*_0_ and *H*_1_^66^.

SEM was conducted to estimate the relationship among all the latent variables, using AMOS 18.0^67,68^. A latent variable “cognitive control” (CC) was derived from CCC and EC. A latent variable, “N-back”, was derived from the performance indices of two N-back tasks (spatial and verbal), and the other latent variable, “working memory span” (WMS), was derived from three span scores of OSpan, RotSpan, and SymSpan. A second-order latent variable, “working memory” (WM), was derived from the latent variables of N-back and WMS. A latent variable, “Gf”, was derived from PRI, WMI, and PSI, and a latent variable “Gc” was derived from VCI. A second-order latent variable, “IQ”, was derived from the latent variables of Gf and Gc to represent the general intellectual ability. We used the maximum likelihood estimation method, which is the most commonly utilized, to select the set of values that maximizes the likelihood of observed covariance^69^.

In order to examine the relationship among intelligence, cognitive control, and working memory, we estimated four models: (1) an overall model with IQ, CC, and WM as the latent variables was estimated to examine the relationship among them; (2) a model with Gc, Gf, and CC as latent variables was estimated to directly examine their relationship, and to examine the relationship among different components of IQ (Gc and Gf) and CC; (3) a model with Gc, Gf, and WM as latent variables was estimated to directly examine their relationship, and to further examine the relationship among different components of IQ and WM; and (4) a model with CC and WM as latent variables was estimated to test the relationship between them. Standardized estimates are presented in all models. Negative error variances were constrained to 0^70,71^. Fisher’s r-to-z transformation was conducted to test the significance of the difference between two correlations coefficients.

Multiple fit measures, including the ratio of chi-square over degrees of freedom (*χ*^2^/*df*), root mean square error of approximation (RMSEA), Tucker Lewis index (TLI), comparative fit index (CFI), and Bayesian information criterion (BIC), were calculated to assess how effectively the models captured the covariance between the variables. In line with previous publications^72,73^, the cut-off criteria used to establish the good fit between the hypothesized model and the observed data were considered acceptable when the χ^2^/df is less than 2, the RMSEA is less than 0.06, and the TLI and CFI are above 0.95. If a pair of variables theoretically correlated to each other and showed a modification indices for the covariance between their error variances greater than 4, the error variances in the model were linked to improve the model fit^74,75^. For the model comparison, chi-square difference was tested. In addition, a BIC difference greater than 2 indicates positive evidence against the model with higher BIC (2–6: positive; 6–10: strong; >10: very strong)^76^.

## Results

The composite scores of the WAIS-IV and the performance scores of the behavioral tasks are shown in Table 1. The mean (and SD) FSIQ score, an estimate of general intellectual ability, was 97.01 (8.07). The mean (and SD) of VCI, PRI, WMI, and PSI were 99.05 (9.49), 93.13 (10.29), 98.78 (9.76) and 100.81 (11.48), respectively. The mean (and SD) of the CCC and the EC were 3.82 (0.62) bps and −144.06 (41.65) ms, respectively. The mean (and SD) performance indices of the spatial and verbal N-back tasks were −0.47 (0.21) and −0.09 (0.13), respectively. In addition, the mean (and SD) of ANL scores was 0.56 (0.23) for OSpan, 0.44 (0.23) for RotSpan, and 0.45 (0.26) for SymSpan.

**Table 1:** Mean, standard deviation (SD), and range for the indices of behavioral tasks and composite scores of the WAIS-IV.

	Mean	SD	Min	Max
*Intellectual ability*				
FSIQ	97.01	8.07	79	117
VCI	99.05	9.49	80	125
PRI	93.13	10.29	73	121
WMI	98.78	9.76	77	122
PSI	100.81	11.48	81	135
*Cognitive control*				
CCC (bps)	3.82	0.62	2.04	5.08
EC (ms)	−144.06	41.65	−82.32	−276.42
*Working Memory*				
Spatial 0-back	0.99	0.02	0.92	1.00
1-back	0.76	0.19	0.17	1.00
2-back	0.51	0.22	0.06	0.99
N-back (2backminus 0back)	−0.47	0.21	−0.92	−0.01
Verbal 0-back	0.95	0.05	0.83	1.00
1-back	0.91	0.11	0.47	1.00
2-back	0.90	0.13	0.38	1.00
3-back	0.87	0.12	0.47	1.00
N-back (3backminus 0back)	−0.09	0.13	−0.53	0.17
OSpan	0.56	0.23	0	1
RotSpan	0.44	0.23	0	1
SymSpan	0.45	0.26	0	1

*Note: CCC*: capacity of cognitive control; *bps*: bits per second; *EC*: executive control; *OSpan*: operational span task; *RotSpan*: rotation span task; *SymSpan*: symmetric span task. *FSIQ*: Full Scale Intelligence Quotient; *VCI*: Verbal Comprehension Index; *PRI*: Perceptual Reasoning Index; *WMI*: Working Memory Index; *PSI*: Processing Speed Index.

### Correlation among the composite scores of the WAIS-IV and all task performance

Correlation coefficients between the composite scores of the WAIS-IV, different measures of cognitive control and working memory, and BF values are shown in Table 2. For the correlation coefficients among the measures within each construct, the FSIQ was significantly and positively correlated to all its composite scores (VCI, PRI, WMI, and PSI) in the WAIS-IV (*rs* = 0.54–0.77, *ps* < 0.001). VCI was significantly correlated to PRI (*r* = 0.31, *p* = 0.002) and to WMI (*r* = 0.28, *p* = 0.004), while PRI was significantly correlated to WMI (*r* = 0.18, *p* = 0.045). WMI was not correlated to PSI (*r* = 0.10, *p* = 0.171, BF < 1/3). The CCC was significantly and positively correlated to EC (*r* = 0.22, *p* = 0.021), indicating that higher CCC was associated with more efficient EC (less negative conflict effect). The spatial N-back was significantly correlated to the SymSpan only (*r* = 0.21, *p* = 0.027), the verbal N-back was not correlated to any other WM measures (*rs* = 0.01–0.10, *ps* > 0.05, BF < 1/3), and the three WM complex spans were significantly and positively correlated to each other (*rs* = 0.34–0.41, *ps* < 0.001).

**Table 2:** Pearson’s correlation coefficients (and Bayes Factor values) among all IQ, CC, and WM measures.

	FSIQ	VCI	PRI	WMI	PSI	CCC	EC	Spatial N-back	Verbal N-back	OSpan	RotSpan
VCI	0.68***	—									
	(>100)										
PRI	0.77***	0.31**	—								
	(>100)	(6.20)									
WMI	0.54***	0.28**	0.18*	—							
	(>100)	(2.72)	(0.34)								
PSI	0.55***	0.05	0.33***	0.10	—						
	(>100)	(0.09)	(11.37)	(0.13)							
CCC	0.39***	0.17	0.28**	0.46***	0.16	—					
	(94.25)	(0.29)	(2.72)	(>100)	(0.25)						
EC	0.30**	0.27**	0.21*	0.12	0.14	0.22*	—				
	(4.66)	(2.11)	(0.57)	(0.16)	(0.20)	(0.69)					
Spatial N-back	0.33***	0.21*	0.19*	0.28**	0.20*	0.32***	0.13	—			
	(11.37)	(0.57)	(0.40)	(2.72)	(0.48)	(8.35)	(0.17)				
Verbal N-back	0.16	0.10	0.14	0.15	0.02	0.10	0.06	0.04	—		
	(0.25)	(0.13)	(0.20)	(0.22)	(0.09)	(0.13)	(0.10)	(0.09)			
OSpan	0.30**	0.13	0.18*	0.43***	0.11	0.36***	0.18*	0.15	0.01	—	
	(4.66)	(0.17)	(0.34)	(>100)	(0.14)	(30.92)	(0.34)	(0.22)	(0.08)		
RotSpan	0.32***	0.11	0.27**	0.27**	0.19*	0.17	0.23*	0.12	0.11	0.41***	—
	(8.35)	(0.14)	(2.11)	(2.11)	(0.40)	(0.29)	(0.85)	(0.16)	(0.14)	(>100)	
SymSpan	0.54***	0.33***	0.49***	0.25**	0.28**	0.36***	0.35***	0.21*	0.10	0.34***	0.46***
	(>100)	(11.37)	(>100)	(1.31)	(2.72)	(30.92)	(21.89)	(0.57)	(0.13)	(15.68)	(>100)

*Note: n* = 88. *p < 0.05; **p < 0.01; ***p < 0.001 (one-tailed). Values below the correlation coefficients represent the corresponding Bayes factor (BF). BF > 100: decisive evidence for the correlation; BF > 3: substantial evidence for the correlation; BF < 1/3: substantial evidence for no correlation; 1/3 ≤ BF ≤ 3: insensitivity in detecting correlation.

For the correlation coefficients between measures of different constructs, we report coefficients that are only relevant to our hypothesis testing. Refer to Table 2 for all the other coefficients. The FSIQ was significantly correlated to CCC, EC, spatial N-back, OSpan, RotSpan, and SymSpan (*rs* = 0.30–0.54, *ps* < 0.01); the VCI was significantly correlated to EC, spatial N-back, and SymSpan (*rs* = 0.21–0.33, *ps* < 0.05); the PRI was significantly correlated to CCC, EC, spatial N-back, OSpan, RotSpan, and SymSpan (*rs* = 0.18–0.49, *ps* < 0.05); the WMI was significantly correlated to CCC, spatial N-back, OSpan, RotSpan, and SymSpan (*rs* = 0.25–0.46, *ps* < 0.01), but not to the EC (*r* = 0.12, *p* = 0.274, BF < 1/3); the PSI was correlated to spatial N-back, RotSpan, and SymSpan (*rs* = 0.19–0.28, *ps* < 0.05), but not correlated to either CCC (*r* = 0.16, *p* = 0.069, BF < 1/3), and EC (*r* = 0.14, *p* = 0.095, BF < 1/3); the CCC was significantly correlated to spatial N-back, OSpan, and SymSpan (*rs* = 0.32–0.36, *ps* < 0.001); the EC was significantly correlated to OSpan, RotSpan, and SymSpan (*rs* = 0.18–0.35, *ps* < 0.05).

### Structural equation modeling results

**Figure 5:**
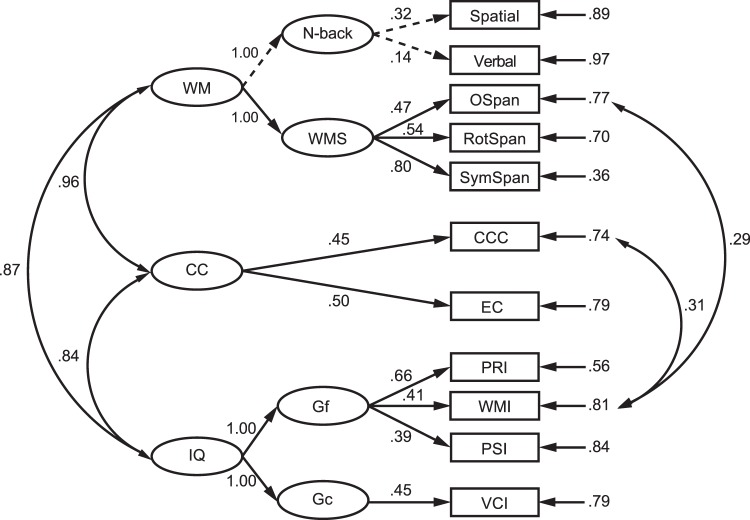
Structural equation model with general intelligence (IQ), cognitive control (CC), and working memory (WM) as latent variables. Double-headed arrows connecting second-order latent variables (ellipses) represent the correlations between constructs. Single-headed arrows from the second-order latent variables to the first-order latent variables (ellipses), and from the latent variables to the manifest variables (rectangles) represent the loadings on the constructs. Single-heading arrows with numbers pointing to the manifest variables represent the error variance of each task. Double-headed lines connecting the error variances represent the correlations between measures. Standardized coefficients of correlations and loadings are presented next to the arrows. Significant paths are displayed by the solid lines (*ps* < 0.05), while nonsignificant paths are displayed by the dashed lines.

**Figure 6:**
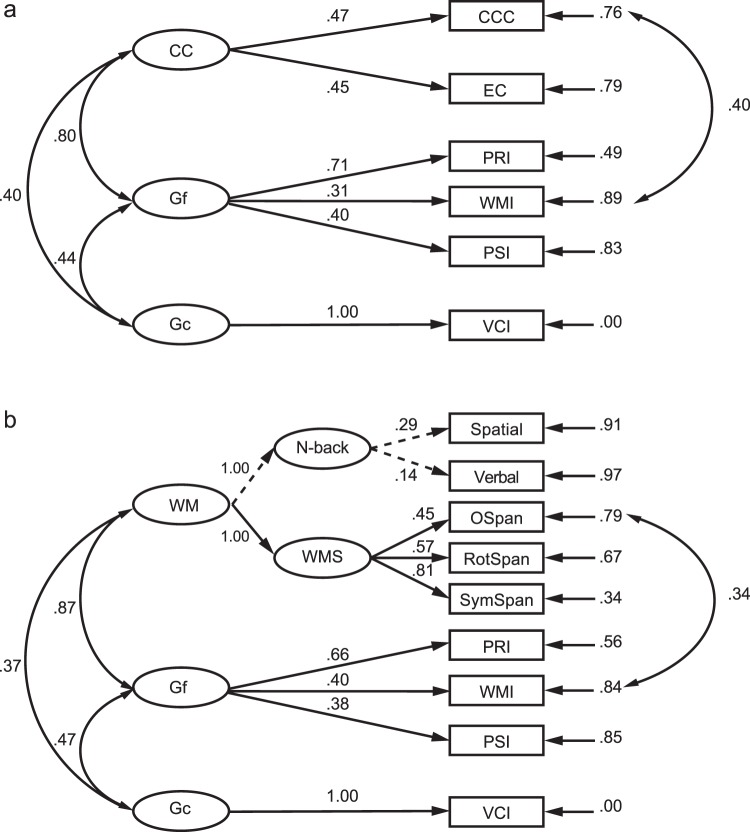
Structural equation models with cognitive control and working memory as latent variables. (**a**) SEM with CC, Gf, and Gc as latent variables. (**b**) SEM with WM, Gf, and Gc as latent variables. Significant paths are indicated by solid lines (*ps* < 0.05), while nonsignificant paths are indicated by dashed lines.

**Figure 7:**
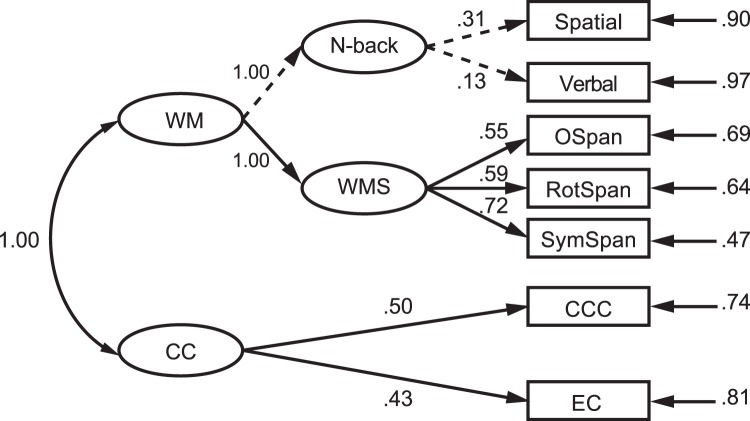
Structural equation model with WM and CC as latent variables. Significant paths are indicated by solid lines (*ps* < 0.05), while nonsignificant paths are indicated by dashed lines.

Table 3 summarizes fit indices of the four models examined, indicating good fit of all the models. Figures 5–7 show the regression weights of the manifested variables to the latent variables, which were significant (*ps* < 0.05) except for the two N-back tasks, suggesting that most of the tasks were able to represent the constructs of interest. All the latent variables were significantly correlated to each other (*rs* = 0.37–1.00, *ps* < 0.05).

**Table 3:** Fit indices for all models.

Model	χ^*2*^ (df)	RMSEA	TLI	CFI	BIC
IQ, CC, and WM	35.33 (39)	0.00	1.04	1.00	156.22
Gc, Gf, and CC	8.12 (7)	0.00	1.02	1.00	70.80
Gc, Gf, and WM	24.09 (25)	0.04	0.95	0.98	113.66
CC and WM	13.83 (13)	0.03	0.98	0.99	80.99

*Note: RMSEA*: root mean square error of approximation; *TLI*: Tucker Lewis index; *CFI*: comparative fit index; *BIC*: Bayesian information criterion.

Figure 5 shows the overall model with IQ, CC, and WM as the latent variables. The error variances of WMI in Gf and CCC in CC, and the error variances of WMI in Gf and OSpan in WMS were linked to improve the model fit. In this model, IQ was strongly correlated to CC (*r* = 0.84, *p* = 0.017) and WM (*r* = 0.87, *p* = 0.021), and CC was strongly correlated to WM (*r* = 0.96, *p* = 0.001), indicating that a large amount of common variance was shared by the three constructs. Fisher’s r-to-z transformation showed that the correlation between IQ and WM was greater than the correlation between IQ and CC, *z* = 1.97, *p* = 0.024 (one-tailed), resulting in an additional 5% of variance explained by WM compared to CC. See the discussion section regarding a potential explanation of this result.

Figure 6a shows the model examining the relationship among Gc, Gf, and CC. In this model, the error variances of WMI in Gf and CCC in CC were linked to improve the model fit, and the negative error variance of VCI in Gc was constrained to 0. CC was significantly correlated to both Gf (*r* = 0.80, *p* = 0.013) and Gc (*r* = 0.40, *p* = 0.041). The correlation between CC and Gf was significantly greater than the correlation between CC and Gc, *z* = 5.21, *p* < 0.001 (one-tailed), suggesting that a greater amount of variance was shared between Gf and CC than between Gc and CC. Additionally, Gf and Gc were positively correlated (*r* = 0.44, *p* = 0.002). Figure 6b shows the model examining the relationship between Gc, Gf, and WM. In this model, the error variances of WMI in Gf and OSpan in WMS were linked to improve the model fit, and the negative error variance of VCI in Gc was constrained to 0. WM was significantly correlated to both Gf (*r* = 0.87, *p* < 0.001) and Gc (*r* = 0.37, *p* = 0.002). The correlation between Gf and WM was significantly greater than the correlation between Gc and WM, *z* = 7.50, *p* < 0.001 (one-tailed), suggesting that a greater amount of variance was shared between Gf and WM than between Gc and WM. In addition, Gf and Gc were significantly correlated (*r* = 0.47, *p* < 0.001), a result consistent with findings from a previous study (Friedman *et al*., 2006). For the model comparison, the difference between the CC-Gf-Gc and WM-Gf-Gc models tested as chi-square difference was not significant (Δ*χ*^2^ = 15.97, Δ*df* = 18, *p* > 0.05). Further, the comparison of the BIC values showed that the difference between these two models was 42.86, with higher BIC (i.e., 113.66) for the WM-Gf-Gc model and lower BIC (i.e., 70.80) for the CC-Gf-Gc model, indicating that the latter model was better than the former model in terms of model fit.

Figure 7 shows the model examining the relationship between CC and WM as latent variables. CC was strongly correlated to WM (*r* = 1.00, *p* < 0.001), indicating a strong link between these two constructs.

The non-significant regression weights of the two N-back tasks to the latent variable “N-back” in all models might be due to the ceiling effect in the verbal N-back task. In this task, the accuracy of each condition in verbal N-back was very high (0-back: 0.95, 1-back: 0.91, 2-back: 0.90, 3-back: 0.87) and the difference of accuracy between conditions of 0-back and 3-back (verbal N-back index) was very small, suggesting that less mental operation might have been involved and that the cognitive load in this task was not adequate to challenge cognitive control. Similarly, we found no correlations between the verbal N-back index and any other WM measures as well as cognitive control and IQ measures. To examine the influence of the verbal N-back index, we removed the manifest variable of verbal N-back in all of the current models in Figs 5, 6b, and 7. Results of these new models showed that N-back loaded significantly onto working memory (*ps* < 0.05).

## Discussion

The significant relationship between cognitive control and intellectual abilities, especially the Gf, suggests that cognitive control is a core component of human intelligence. The two measures used to estimate cognitive control, the EC and the CCC, tap on participants’ ability to simultaneously select and prioritize visual inputs that are behaviorally relevant and to coordinate mental operations under uncertainty. This core component is also involved in the performance of subtests of the WAIS-IV, especially in those tapping on Gf such as Matrix Reasoning which requires examinees to select a reasonable geometric pattern from a set of options to complete a matrix or a series, giving a measure of classification and spatial ability, simultaneous processing, and perceptual organization^77–79^. Further, the significant zero-order correlations found between the CCC and the composite scores of the Gf (including PRI and WMI subscales), as well as the significant correlation between the EC and the PRI, also favor the explanation of the involvement of cognitive control in human intelligence. The significant correlation found between the latent variables of CC and of Gc (VCI subscale) could instead be pointing at the involvement of the abstract reasoning processes in the Similarities subtest in VCI^53^. The need of a coordination of thoughts and actions to perform the IQ tests explains the association between CC and IQ, supporting our hypothesis that cognitive control serves as a core component of intellectual abilities.

The significant correlations among CC, WM, and IQ suggest that cognitive control may be the common factor involved in all of these constructs. We found a significant correlation between WM and IQ, especially the Gf, which is consistent with existing literature^22,28,80^ indicating that working memory capacity is a primary predictor of general intelligence (*g*), if not essentially isomorphic^81^. The significant correlation between the latent variables of CC and WM indicates that these two constructs share the core component. We speculate that that the shared component between WM and CC is the coordination of thoughts and actions, i.e., cognitive control, rather than the memory storage component. Specifically, the performance of the MFT-M involves the sensory or iconic memory which is automatic, high-capacity, and short-lived^82^, and this component emerges at a lower level of information processing and is theoretically distinct from higher-level mental operation. Further, participants using the grouping search algorithm do not need to store information of previous outcomes of sampling in working memory, which keeps the WM loads constant across MTF-M conditions. In addition, the majority size is 3, which is much smaller than the working memory capacity (WMC)^20^. Thus, the CCC measured by our task should not be limited and impacted by WMC. Thus, the shared component between these two constructs should be independent from memory storage, favoring our hypothesis that cognitive control is the core component. Regarding the significant correlation between WM and the Gf, it is in line with the central executive account of Gf ^28,83–85^, which proposes that the relationship between working memory and Gf may be related to the functioning of the central executive process. On a similar line of evidence, in our previous study^45^ we proposed that executive control is one of the attentional functions whose interplay underlies cognitive control, and our current results suggest that the strong link between working memory and Gf may be related to the involvement of cognitive control in these constructs. Unsurprisingly, the stronger correlation between WM and Gf compared to the correlation between CC and Gf (i.e., the additional 5% of variance explained) might be due to simultaneous presence of the memory component and of the cognitive control component in the WM tasks.

We found that CC was significantly associated with Gf in term of latent variables in our models. However, the patterns of the zero order correlation for the two measures of cognitive control, the CCC and the EC, in relation to the indices of Gf including PRI, WMI, and PSI were different. In the current study, we found that both the CCC and EC were positively correlated to the PRI. This result may indicate that both capacity and processing efficiency play an important role in perceptual reasoning of fluid intelligence^86,87^. Further, the CCC was significantly correlated to the WMI subscale, while the EC was not. A potential explanation for this pattern may reside in the difference between the capacity of cognitive control and its processing efficiency. The CCC measures the upper limit of mental operation involved in cognitive control^46,47^, whereas the EC, assessed by the ANT-R, represents the processing efficiency of cognitive control in a unit of time to a fixed amount of information^44^. The WMI was significantly correlated to CCC because it measures the capacity of working memory, which is greatly related to the manipulation of information^29^. Surprisingly, although we argue that cognitive control is for the coordination of mental operation, we found no evidence for the correlation between both CCC/EC and the PSI. This subscale of the WAIS was designed according to the processing efficiency theory^88^, which suggests that processing efficiency represents the information-processing rate required by a task. The little involvement of thought processing in PSI may explain our negative result because, as shown by previous studies, a strong relationship between processing speed and fluid intelligence can be found only in condition of high mental demands^22,89,90^.

The existence of a link between CC and IQ is also supported by the common involvement of the fronto-parietal network found in studies investigating cognitive control^47,58,91–100^ and intellectual activity^101–106^. In addition to the psychometric studies, the neuromechanism of intelligence has recently attracted increased attention. Even though alternative theories are currently being explored, a great extent of interest has been directed towards a network of regions in the frontal and parietal lobes that consistently co-activates across domains involved in intellectual activity. For example, a recent Parieto-Frontal Integration Theory (P-FIT) has identified a distributed brain network supporting human intelligence, especially its fluid component, including the anterior cingulate cortex, the inferior and superior parietal lobule, and the dorsal prefrontal cortex^102,103,107,108^. Further, fluid intelligence has been associated with a multiple-demand system^8,9^ residing in somewhat overlapping regions with those proposed by the P-FIT model. This “intelligence network” greatly overlaps with the cognitive control network, and includes the regions of the anterior insular cortex, the anterior cingulate cortex, the frontal eye field and the intraparietal sulcus^47,109,110^, strengthening the conclusion that intellectual activity is supported by cognitive control processes with the cognitive control network as its substrate.

Our cognitive control model of human intelligence emphasizes the mental operations for the coordination of thoughts and actions in intelligent behaviors, which is not fundamentally opposed to the previous models of intelligence, such as the *g* factor theory, the Gf-Gc theory, the triarchic theory, or the PASS theory. For instance, although the *g* factor underlies all cognitive abilities and has been regarded as the most fundamental factor (the apex) of the hierarchical structure of intelligence^3,90^, its nature remains unclear. Our model attempts to link the nature of *g* factor to cognitive control, an essential intellectual component that may be added to the psychometric measurements of intelligence. Similarly, we propose that cognitive control is a core element in the three components in the triarchic theory and in the four mental processes included in the PASS theory. Both theories have been proposed as alternative models to general intelligence^16,111^, attempting to define the important aspects of human intellectual competence by identifying independent but interactive cognitive processes/components. However, whether a common component across these processes/components exists was not emphasized. According to the definitions and the manipulations involved in the PASS tests such as the PASS Reading Enhancement Program (PREP)^112^, planning would be the most complex process that includes all the other three processes to solve problems^113^. The process of planning involves dynamic coordination of thoughts and actions, together with an evaluation of the behavioral outcome, to carry out the goal-directed behaviors, which should involve the process of cognitive control as the core. The evidence that the key regions of planning are located at the prefrontal cortex^114^, which is also one of the substrates underlying cognitive control, may further support our argument.

As a core component in Gf, cognitive control could be used to explain a well-known phenomenon of goal-neglect^115^, which has been shown in populations and individuals with lower Gf, such as patients with damage in the frontal lobe^116,117^ and neurotypical controls^115,118–120^. It refers to participants’ performance failure on a specific task due to limited capacity of information processing although they are able to correctly recall the task requirement. Goal neglect has been shown to be greatly influenced by task complexity^115^ and the ability to convert complex requirements into effective attentional episodes or cognitive segmentation^119,121,122^. Based on these accounts, an efficient way to improve cognitive performance is to (1) dissect an unstructured and chaotic problem/goal into simpler sub-problems/sub-goals; and (2) only a sub-goal of behavior needs to be achieved in each attentional episode or each piece of cognitive segmentation. The essence of this strategy is to reduce the load of cognitive control for the coordination of thoughts and actions between attentional episodes. Even within each attentional episode, cognitive control is necessary to achieve subgoal-directed behavior. Therefore, this strategy of cognitive segmentation is consistent with our information theory account of cognitive control that is to reduce uncertainty^44^.

In the current study, we showed the correlation between CCC and IQ in a homogenous group of young participants with mean IQ scores around 100, and standard deviations of maximally 11 IQ points. In another study of our group^123^, a significant correlation between CCC and IQ was found (*r* = 0.55, *p* = 0.003) in a group of individuals (n = 27) with higher mean IQ scores (mean = 124.56, SD = 12.70), indicating that our model is also valid in neurotypical groups with higher IQ scores. In previous studies on goal neglect^115,119^, major performance failures of tests were restricted to participants in the lower range of IQ scores, suggesting that this association is also true in individuals with lower IQ. However, further studies are needed to test the validity of our cognitive control model of human intelligence for neurotypical groups with low Gf. Additionally, our model seems to be able to explain previously shown deficits in the coordination of mental operations in individuals with mental retardation^124,125^, neurodevelopmental^126–128^, and psychiatric disorders^129–132^, resulting from a functional deficit of the areas within the cognitive control network^133^.
